# Inhibition of DNMT3B expression in activated hepatic stellate cells overcomes chemoresistance in the tumor microenvironment of hepatocellular carcinoma

**DOI:** 10.1038/s41598-023-50680-6

**Published:** 2024-01-02

**Authors:** Yeonhwa Song, Namjeong Kim, Jinyeong Heo, David Shum, Taemoo Heo, Haeng Ran Seo

**Affiliations:** 1 Advanced Biomedical Research Laboratory, Institut Pasteur Korea, 16, Daewangpangyo-ro 712 beon-gil, Bundang-gu, Seongnam-si, Gyeonggi-do 13488 Republic of Korea; 2 Screening Discovery Platform, Institut Pasteur Korea, 16, Daewangpangyo-ro 712 beon-gil, Bundang-gu, Seongnam-si, Gyeonggi-do 13488 Republic of Korea

**Keywords:** Cancer therapy, Molecular biology

## Abstract

Hepatocellular carcinoma (HCC) is a complex disease associated with a plethora of environmental and genetic/hereditary causative risk factors, more so than other oncological indications. Additionally, patients with HCC exhibit fibrosis, cirrhosis, and liver-related disease. This complicated etiology can affect the disease course and likely contributes to its poor prognosis. In this study, we aimed to improve HCC therapy by evaluating combination treatment using anti-cancer and anti-fibrosis drugs via identification of novel anti-fibrosis drugs. We performed high-throughput screening of 10,000 compounds to identify hepatic fibrosis inhibitors through morphometry analysis of multicellular hepatic spheroid (MCHS) models and identified CHIR-99021 as a candidate anti-fibrotic drug. Treatment with CHIR-99021 induced loss of cell–cell interactions and suppression of extracellular matrix-related protein expression via reprogramming of hepatic stellate cell (HSC) activation in MCHSs. In particular, CHIR-99021 regulated DNMT3B expression only in activated HSCs. Moreover, CHIR-99021 markedly improved the efficacy of sorafenib in HCC- multicellular tumor spheroids in vitro and through induction of apoptosis by decreasing DNMT3B expression in vivo. In summary, these findings suggest that targeting HSC reprogramming by attenuation of DNMT3B expression in the tumor environment might represent a promising therapeutic strategy for liver fibrosis and HCC.

## Introduction

Hepatocellular carcinoma (HCC) is one of the most frequently occurring types of cancer and is associated with a high mortality rate worldwide^1^. The highest incidence rates of HCC occur in Eastern Asia and Sub-Saharan Africa^2,3^. Recently, the incidence of HCC has been consistently increasing in the Western world^4^. The lethality of HCC can be attributed to its therapeutic resistance. Nevertheless, angiogenesis inhibitors and immune checkpoint inhibitors are currently used to eliminate HCC in liver cancer therapy^5^. However, neither approach is entirely effective against HCC^6^.

In recent years, the number of patients with non-viral HCC, including nonalcoholic steatohepatitis (NASH)/nonalcoholic fatty liver disease-related HCC, has been increasing^7^. The histopathology of liver cancer involves fibrosis, cirrhosis, and liver-related metabolic pathophysiology. Accordingly, drug-induced properties of interest include anti-fibrotic, anti-inflammatory, and metabolic regulatory effects.

Recently, tevacizumab and atezolizumab, a first-in-class combination, showed an increase in overall survival and prolonged disease-free survival compared with sorafenib in patients with HCC^8^. Because of this combination of an immuno-oncology agent and an angiogenesis inhibitor, drug discovery strategies are undergoing a shift in preference from monotherapy to combination regimens for HCC therapy^9,10^. In this study, we evaluated combination treatment with anti-cancer and anti-fibrotic drugs for HCC therapy because we found an increase in anti-cancer efficacy through the alleviation of tissue fibrosis.

To identify novel anti-fibrotic drugs, we utilized multicellular hepatic spheroid (MCHS) models composed of HCC cells and hepatic stellate cells (HSCs)^11^ because activated HSCs play a pivotal role among stromal cells in liver fibrosis progression and HCC chemoresistance through the accumulation of extracellular matrix-related molecules^12^. Hence, inducing reversion of human HSC activation might represent a promising therapeutic strategy for liver fibrosis and HCC^13,14^.

CHIR-99021 (Laduviglusib) acts as an agonist of Wnt signaling by inhibiting GSK3β activity and is suggested to be a useful therapeutic for the treatment of insulin resistance in type 2 diabetes^15^.

CHIR-99021 is commonly used to maintain self-renewal and pluripotency of embryonic stem cells^16,17^. Human primary hepatocytes were reprogrammed towards hepatic progenitor cells by treatment with CHIR-99021^18^. CHIR-99021 has also been shown to aid in inhibition of the endothelial-mesenchymal transition (EMT)^19,20^. Here, we hypothesized that CHIR-99021 treatment could enhance the transdifferentiation of HSCs from a fibrogenic phenotype to a quinescent phenotype in a fibrogenic environment.

Epigenetic mechanisms have been increasingly recognized as playing a central role in HSC differentiation^21,22^. Among epigenetic enzymes, such as DNA methyltransferases (DNMTs), histone methyltransferases, and histone deacetylases, targeting of DNMTs has demonstrated their essential roles in organ fibrosis^23^. DNMTs found in mammalian cells include DNMT1, DNMT3A, and DNMT3B. DNMT3A and DNMT3B exhibit de novo DNA methylation activity, whereas DNMT1 plays a central role in preserving DNA methylation patterns through cell division^24,25^.

In this study, the anti-fibrogenic effects of CHIR-99021 on key aspects of HSC reprogramming were observed in cultured HSCs and MCHSs. Mechanistically, we investigated the effects of CHIR-99021 on epigenetic modification in activated HSCs. Using CHIR-99021, we investigated the effects of combining an anti-fibrotic agent and an angiogenesis inhibitor on HCC therapy in vitro using hepatic multicellular tumor spheroids (MCTS) and in an in vivo system. Based on our observations, we also discuss future directions for therapeutic opportunities.

## Materials and methods

### Cell culture

The human liver cancer cell lines Huh7 and PLC/PRF/5 were obtained from the Korean Cell Line Bank (Seoul, Korea) and maintained in Roswell Park Memorial Institute medium (RPMI 1640; Welgene, Daegu, Korea) containing 10% fetal bovine serum (FBS; Gibco, Grand Island, NY, USA) and 1% penicillin–streptomycin (P/S; Gibco). The LX2 human HSC line was purchased from Merck Millipore (Darmstadt, Germany) and maintained with Dulbecco’s modified Eagle’s medium (DMEM; Welgene) containing 2% FBS and 1% P/S. Another human HSC line, LI90, was purchased from JCRB Cell Bank (Japan) and cultured in DMEM containing 10% FBS and 1% P/S. An HCC cell line, HepG2, and the WI38 human fibroblast cell line were obtained from ATCC (Manassas, VA, USA). These cell lines were maintained in minimum essential media (Welgene) supplemented with 10% FBS and 1% P/S. Human umbilical vein endothelial cells (HUVECs) were purchased from PromoCells (Heidelberg, Germany) and cultured in endothelial basal medium with supplementary reagents from PromoCells. All cells were maintained at 37 °C in a humidified incubator with 5% CO_2_.

### Generation of MCHSs

To generate MCHSs, the method in previous study was applied^11^. Briefly, cells were seeded at a density of 6 × 10^3^ cells/well in 96-well, round-bottom, ultra-low-attachment microplates (Corning Life Sciences, Amsterdam, Netherlands). For MCTS generation, HCC cells were seeded at a density of 3.3 × 10^3^ cells/well, and LX2 and WI38 cells and HUVECs were seeded at a density of 0.9 × 10^3^ cells/well/cell line together in 96-well, round-bottom, ultra-low-attachment microplates. The plates were incubated for 3 days at 37 °C in a humidified incubator with 5% CO_2_. After 3 days, anti-fibrosis drugs were added, and the cells were incubated for an additional 2 days. Spheroids composed of 4.2 × 10^3^ HCC cells and 1.8 × 10^3^ HSCs were created to investigate interactions between HCC cells and HSCs. The spheroids were incubated in ultra-low-attachment microplates for 3 days.

### Compounds

Obeticholic acid (OCA, SML3096, Sigma-Aldrich, St Louis, MO, USA), CHIR-99021 (Selleck Chemicals, Houston, TX, USA), CHIR-98014 (Selleck Chemicals), GW4064 (G5172, Sigma-Aldrich), SB-216763 (S3442, Sigma-Aldrich), TWS-119 (SML1271, Sigma-Aldrich), Tideglusib (SML0339, Sigma-Aldrich), SB-415286 (S3567, Sigma-Aldrich), and nanaomycin A(sc-396527, Selleckchem) were used in the study.

### Western blot analysis

Cell pellets were collected by centrifugation and lysed in a lysis buffer (Thermo Fisher Scientific, MA, USA). The supernatants were collected by centrifugation at 12,000 rpm for 20 min. The amount of protein was determined using a bicinchoninic acid method according to the manufacturer’s instructions (Thermo Fisher Scientific). Equal amounts of protein were separated on 8% or 10% sodium dodecyl sulfate–polyacrylamide gels. After electrophoresis, the proteins were transferred onto a nitrocellulose membrane (Pall, Port Washington, NY, USA). Before hybridization with antibodies, the membranes were cut depending on the size of protein. The membranes were blocked with 5% skim milk (BD Bioscience, Franklin Lakes, NJ, USA) for 30 min at room temperature (R.T.). After blocking, the membranes were immunoblotted with the following specific primary antibodies: E-cadherin (ab40772, 1:200), N-cadherin (ab76057, 1:500), human alpha-smooth muscle actin (α-SMA, ab32575, 1:3000), Vimentin (ab8978, 1:3000), Cleaved-caspase 3 (ab2302, 1:1000), and p-GSK3β (Y219, ab75745, 1:000) purchased from Abcam (Cambridge, MA, USA); and Snail (3879 s, 1:1000), Smad2/3 (3102, 1:1000), p-Smad2 (Ser465/467) (3108, 1:1000), p-Smad3 (Ser423/425) (9520, 1:1000), GSK3β (9315 s, 1:1000), p-GSK3β (S9, 9336 s, 1:1000), DNMT1 (5032 s, 1:1000), DNMT3A (3598 s, 1:1000), and DNMT3B (57868 s, 1:1000) purchased from Cell Signaling Technology (Danvers, MA, USA). All primary antibodies were incubated for 16 h at 4 °C. After washing, the blots were incubated with corresponding anti-rabbit and anti-mouse IgGs conjugated with horseradish peroxidase (Cell Signaling Technology) for 1 h. Immunoreactive proteins were detected using an enhanced chemiluminescence reagent (Thermo Fisher Scientific). β-actin was purchased from Sigma-Aldrich and was used as a control for each sample.

### High-throughput screening

A library of 9695 compounds consisting of FDA-approved drugs was assembled from the following sources: Selleck Chemicals, bioactive drugs from the TargetMol database (Wellesley, MA, USA), the Library of Pharmacologically Active Compounds (St Louis, MO, USA), NIH-Clinical Drugs (Bethesda, MA, USA), the Prestwick Chemical Library (Washington, DC, USA) Microsource (Washington, DC, USA), and Tocris Bioscience (Avonmouth, Bristol, UK). The compounds were screened in the MCHS model at a final concentration of 1 µM in 0.5% (v/v) dimethyl sulfoxide (DMSO, Sigma-Aldrich). All cells were seeded at a density of 6 × 10^3^ cells/well into 96-well, round-bottom, ultra-low-attachment microplates. The plates were then incubated at 37 °C in a humidified atmosphere of 5% CO_2_ for 3 days. To test the compounds, a 2-μl sample of each compound was transferred into an intermediate 384-well polypropylene plate (Greiner Bio-one, Monroe, NC, USA) using a liquid handler (Apricot Personal Pipettor; Apricot Design, Covina, CA, USA). The compounds were mixed with 78 μl of complete medium per well. Subsequently, a 20-μl sample of each compound was dispensed into each well of a 96-well assay plate. The plates were then incubated at 37 °C in a humidified atmosphere of 5% CO_2_ for 2 days. OCA was added as a low positive control into each assay plate at its IC_50_, and 0.5% DMSO (v/v) was used as a high control. After 5 days, spheroid images were acquired using a high-content screening system. The spheroid size was measured using a self-developed algorithm. Compounds were selected using a threshold based on 3σ (standard deviations) from the IC_50_ of OCA.

### MCHS staining

After generating MCHSs for 3 days, the spheroids were treated with 0.5 µM CHIR-99021 for an additional 2 days and then collected and transferred to 384-well microplates (781091, Greiner Bio-one). The spheroids were fixed with 4% paraformaldehyde (PFA, Biosesang, Seoul, Korea). For F-actin staining, spheroids were incubated with Alexa Fluor 488 phalloidin (A12379; Invitrogen, Eugene, OR, USA) for 2 h at R.T. After washing three times with Dulbecco's phosphate-buffered saline (DPBS), spheroids were incubated with Hoechst33342 (H3570; Invitrogen) in DPBS for 10 min at R.T. After three washes with DPBS, images were obtained with an Operetta HTS system (Perkin-Elmer, Waltham, MA, USA).

### Anti-fibrosis compound validation in a two-dimensional (2D) model

Anti-fibrosis 2D validation assay were established in previous study^11^. HSCs (LX2 cells) were seeded at 2.5 × 10^3^ cells/well in 384-well microplates and incubated for 16 h at 37 °C in a humidified incubator with 5% CO_2_. LX2 cells were activated with 20 ng/ml recombinant human TGF-β1 (100-21C, Peprotech, Cranbury, NJ, USA), and the test compounds were added for 48 h simultaneously. After incubation, the cells were fixed with 4% PFA for 10 min at R.T. and incubated with F-actin and Alexa Fluor 633 Phalloidin (A22284; Invitrogen) for 2 h at R.T. After three washes with DPBS, the cells were incubated with Hoechst33342 with DPBS for 10 min at R.T. After washing with DPBS, images were obtained using an Operetta HTS system and analyzed using Harmony software (Perkin-Elmer).

### DMNT3B activity

LX2 cells were treated with 0, 0.5 or 1uM of CHIR-99021 for 48 h and nuclear were extracted according to the manufacturer’s instruction (Abcam, Ab113474). DNMT3B activity assay was performed according to the manufacturer’s instruction (Abcam, ab113471) using 30ug of each samples.

### Short interfering RNA (siRNA) transfection

siRNA probes were designed by and purchased from Dharmacon (Lafayette, CO, USA) and Bioneer (Daejeon, Korea). LX2 cells were seeded, and the medium was replaced with Opti-MEM (Gibco) without FBS and antibiotics when the cell density reached 40–50%. The cells were transfected with the four siRNAs targeting DNMT3B (siDNMT3B) and scrambled siRNA for 24 h using Lipofectamine® 3000 (Invitrogen).

### Xenograft study

Huh7 cells (5 × 10^6^ cells/mouse) were transplanted following resuspension in Matrigel (matrix growth factor reduced; BD Biosciences) and orthotopically injected into 5-week-old male BALB/c nude mice (Central Lab. Animal, Inc., Seoul, Korea)^26^. The mice were kept in a laboratory animal facility with a constant temperature of 20 °C ± 2 °C and relative humidity of 50% ± 10% under a regular light–dark cycle. At 2 weeks post-injection, the mice were randomly divided into four groups (5 mice per group): (1) control (saline), (2) 10 mg/kg body weight (mpk) sorafenib, (3) 15 mpk CHIR-99021, and 4) a combination group with 10 mpk sorafenib + 15 mpk CHIR-99021. Each compound or combination was then administered for 2 weeks by intraperitoneal injection. During the experimental period, the body weight and tumor size were measured three times a week using calipers. Tumor volumes were determined using the following formula: (L × I^2^)/2, where L = tumor length and I = tumor width. After mice were sacrificed, the tumor tissues were lysed for protein detection, and the blood was analyzed to detect liver toxicity.

### Statistical analysis

All experiments were performed in duplicate. The results are expressed as the mean ± standard deviation. Statistical analyses were performed using Student’s t-test.

## Results

### Interaction between HSCs and HCC cells facilitates MCHS model compactness

In our previous study, we found that interactions between HSCs and HCC cells produced a myofibroblast-like phenotype and increased collagen I expression in HSCs in a three-dimensional (3D) culture condition. Here, we aimed to utilize MCHS models composed of HCC cells (Huh7, HepG2, and PLC/PRF/5 cells) and HSCs (LX2 and LI90 cells) to target reprogramming of HSCs from a fibrogenic phenotype to quiescent HSCs in a fibrogenic environment.

**Figure 1:**
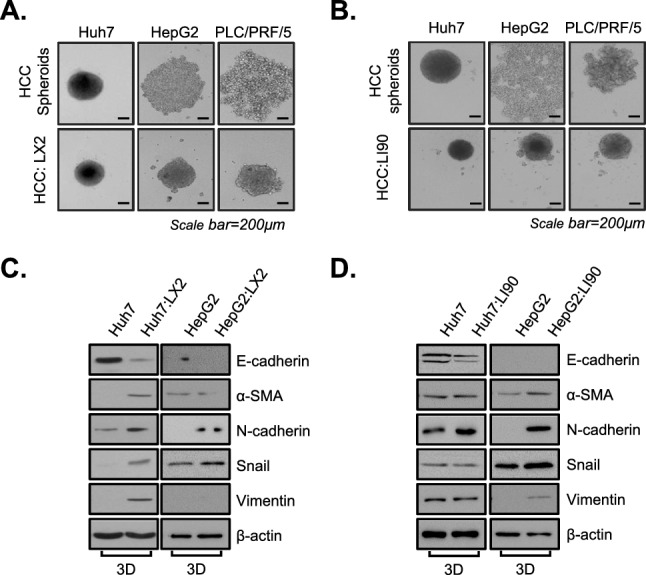
Interaction between HSCs and HCC cells in MCHSs. (**A**) The compactness of spheroids composed of HCC, Huh7, HepG2, and PLC/PRF/5 cells, with or without LX2 HSCs, was observed. (**B**) The compactness of spheroids composed of HCC, Huh7, HepG2, and PLC/PRF/5 cells, with or without LI90 HSCs, was observed. (**C**) Spheroids composed of Huh7 cells and HepG2 cells with or without LX2 cells were examined to detect fibroblast-related proteins, including E-cadherin, α-SMA, N-cadherin, Snail, and Vimentin. (**D**) Spheroids composed of Huh7 cells and HepG2 cells, with or without LI90 cells, were examined to detect fibroblast-related proteins, including E-cadherin, α-SMA, N-cadherin, Snail, and Vimentin. β-actin was used as a control.

HepG2 and PLC/PRF/5 cells loosely bound to each other and failed to show strong cell cohesion during spheroid assembly. However, we clearly observed a profound enhancement of compactness in spheroids containing HSCs and HCC cells relative to those with only HCC cells (Fig. 1A, B).

To determine whether HSCs in MCHSs have fibroblast-like properties, we investigated E-cadherin, N-cadherin, α-SMA, Snail, and Vimentin expression in lysates from MCHSs with both HSCs and HCC spheroids.

In MCHSs with HSCs, expression of E-cadherin, as an epithelial cell marker, was rarely detected, whereas expression of mesenchymal cell markers, including N-cadherin, α-SMA, Snail, and Vimentin, was increased compared with that in HCC spheroids (Fig. 1C, D). These results indicate that HSCs exhibit a myofibroblast-like phenotype and play pivotal roles in the EMT in MCHSs.

### Drug screening for reversion of HSC activation using multicellular spheroids

**Figure 2:**
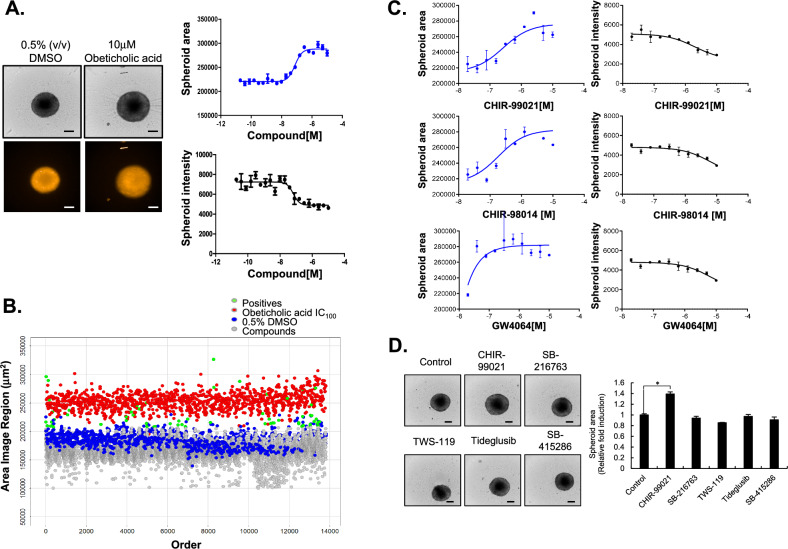
Drug screening of anti-fibrotic compounds in MCHSs. (**A**) Size and Huh7.5-RFP intensity of MCHSs treated with 0.5% DMSO (negative control) or 10 µM OCA (positive control). (**B**) The effects of 9695 drugs on MCHS size were evaluated. (**C**) The MCHS size was evaluated after treatment with different concentrations of CHIR-99021, CHIR-98014, and GW4064 starting at 10 µM. (**D**) Other GSK3β inhibitors in the compound library, such as SB-216763, TWS-119, Tideglusib, and SB-415286 (1 µM) were used to treat MCHSs. The spheroid size was calculated.

In our previous study, we showed that an increase in spheroid size caused by the loss of tight cross-linking between cells because of a lack of fibrosis-related proteins is an appropriate morphometric signature of reversal of liver fibrosis in MCHSs^11,12,27^. Therefore, we performed drug screening to target HSC activation using MCHSs consisting of LX2 and Huh7 cells. We detected alterations in MCHS size and the intensity of red fluorescent protein (RFP) signals in all cells of the MCHSs. The positive and negative controls included 10 µM OCA, which is effective for the treatment of NASH with liver fibrosis, and 0.5% DMSO, respectively. OCA treatment resulted in an increase in MCHS size (from 215.871 to 286.483 µm^2^) and a decrease in RFP intensity (Alexa546: from 6.997 to 4.285) in MCHSs (Fig. 2A, Supplementary Fig. S1A).

We prepared a compound library composed of 9,695 FDA-approved drugs with known molecular targets to identify novel drugs that could revert HSC activation. Compounds were screened at an initial concentration of 1 µM to determine whether they could increase MCHS size similar to the effect of 10 µM OCA. We screened all compounds in duplicate to confirm the reproducibility of the observed effects. The screening identified 20 positive compounds (HITs) (Fig. 2B, Table 1). Next, dose–response studies were performed to identify the compound among the screened drugs that was most effective in preventing liver fibrosis using MCHSs consisting of HSCs and HCC cells.

**Table 1:** The list of HIT compounds from screening.

Compound name	Area (µm^2^)		Intensity		Analysis
	Min	Max	Min	Max	
Obeticholic acid	215,670	283,973	4794	2989	High priority
CHIR-99021	213,761	292,034	2911	5987	High priority
CHIR-98014	215,857	308,126	2862	5193	High priority
GW 4064	216,670	301,007	2879	4056	High priority
DORAMECTIN	218,339	275,305	3167	5108	Low priority
Ch 55	204,184	284,740	4334	8856	Low priority
BMS 649	203,834	266,669	4720	6491	Low priority
CD 1530	173,535	259,662	5233	7466	Low priority
TTNPB	234,690	281,054	5503	6795	Low priority
Bexarotene	243,349	282,992	5148	6681	Low priority
ABT 724 trihydrochloride	207,427	244,103	4640	6015	Inactive
Naproxen sodium	205,662	252,185	3121	5574	Inactive
Topiramate	200,229	232,065	3461	5389	Inactive
PIRACETAM	206,765	226,420	4398	6190	Inactive
Sulindac	192,002	235,173	4517	6055	Inactive
Bosutinib (SKI-606)	210,267	234,489	4879	13,636	Inactive
CP 673451	195,122	254,811	5966	19,603	Inactive
Ginkgolide B	206,227	232,355	4305	5776	Inactive
SB 239063	201,532	236,077	4530	6253	Inactive
Lonidamine	198,253	245,470	4339	5944	Inactive

Among the HITs, CHIR-99021 (Laduviglusib, from 213.761 to 292.034 µm^2^) and CHIR-98014 (from 215.857 to 308.126 µm^2^), which are GSK3β inhibitors, and GW4064 (from 219.805 to 270.577 µm^2^), which is an FXR agonist, produced prominent increases in MCHS size in a dose-dependent manner (from 0.1 µM) relative to other HIT compounds (Fig. 2C, Supplementary Fig. S1). Other GSK3 inhibitors in the compound library, such as SB216763, TWS119, Tideglusib, and SB415286, were not selected as HITs because these compounds could not significantly alter MCHS size similar to the effect of CHIR-99021 (Fig. 2D). Here, we selected CHIR-99021 as the most effective drug for the reversion of activated HSCs.

### CHIR-99021 depolarizes HSCs in a fibrotic environment

**Figure 3:**
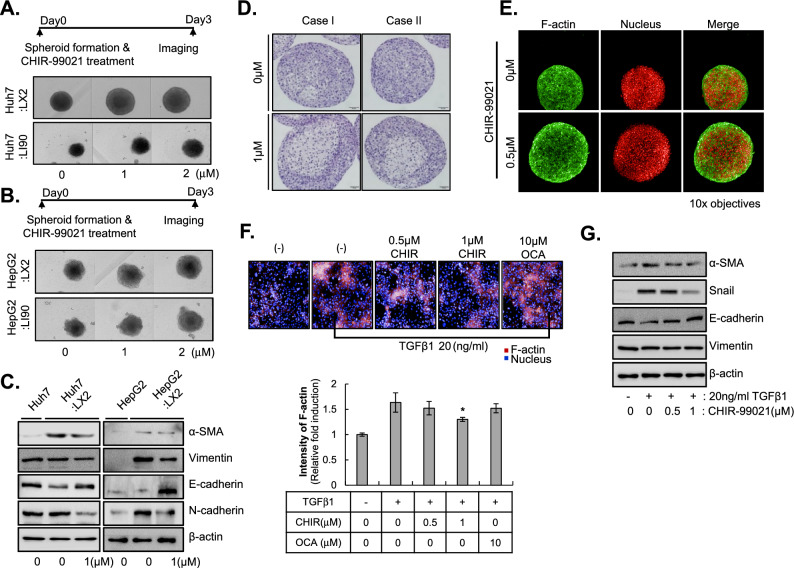
Anti-fibrotic properties of CHIR-99021 in HSCs. (**A**,**B**) The size of MCHSs composed of Huh7 (**A**) or HepG2 cells (**B**) with or without LX2 or LI90 cells was examined after treatment with 1 or 2 µM CHIR-99021. (**C**) EMT-related markers, including α-SMA, Vimentin, E-cadherin, and N-cadherin, were examined in spheroids containing Huh7 (left) or HepG2 cells (right) with or without LX2 cells after treatment with 1 µM CHIR-99021. (**D**) Hematoxylin and eosin staining of MCHSs after treatment with 1 µM CHIR-99021 for 24 h. (**E**) F-actin staining of MCHSs after treatment with 0.5 µM CHIR-99021. (**F**) Stress fibers were measured in 2D cultures of LX2 cells after treatment with 20 ng/ml TGF-β 1 with or without CHIR-99021 (0.5 or 1 µM). OCA was used as a control. Images (upper) were obtained with an Operetta HTS system, and the F-actin intensity was calculated using Harmony software (lower). (**G**) In the 2D system, LX2 cells were treated with 20 ng/ml TGF-β1 with or without 0.5 or 1 µM of CHIR-99021 to detect EMT-related proteins.

Next, we explored the effect of CHIR-99021 on depolarization of HSCs using Huh7 MCHSs containing LX2 cells or LI90 cells. We found that CHIR-99021 treatment produced significant increases size of MCHS in a dose-dependent manner (Fig. 3A). When HepG2 cells were used instead of Huh7 cells, we detected effects similar to those of CHIR-99021 on MCHSs consisting of HSCs and HCC cells (Fig. 3B). Because the EMT leads to upregulation of mesenchymal cell markers, we also measured α-SMA, N-cadherin, and Vimentin expression in MCHSs grown with HSCs and HCC cells after CHIR-99021 treatment to precisely characterize the EMT. α-SMA, N-cadherin, and Vimentin expression levels were significantly decreased after CHIR-99021 treatment, but E-cadherin expression was increased (Fig. 3C).

Hematoxylin and eosin staining was performed to investigate the architectural changes induced by CHIR-99021 treatment in MCHSs consisting of HSCs and HCC cells. Interestingly, addition of 1 µM CHIR-99021 to MCHSs for 24 h increased the spheroid size relative to that of DMSO-treated MCHSs, and we observed loss of cell–cell interactions from the center of MCHSs (Fig. 3D). Fluorescence staining of F-actin also indicated a reduction in the compactness of F-actin fibers in the center of MCHSs as the size was increased by 1 µM CHIR-99021 treatment (Fig. 3E).

To confirm the potential efficacy of CHIR-99021 in reprogramming activated HSCs, we conducted cellular phenotype-based assays. Increased production of F-actin stress fibers is associated with HSC activation when HSCs are stimulated with TGF-β1. TGF-β1 treatment increased the intensity of cytoplasmic F-actin staining in LX2 cells. We measured the effects of CHIR-99021 on TGF-β1-induced HSC activation using cellular phenotype-based assays. As expected, 1 µM CHIR-99021 inhibited F-actin stress fibers after treatment of LX2 cells with TGF-β1, with an efficacy comparable to that of 10 µM OCA, which served as our positive control (Fig. 3F). When 1 µM CHIR-99021 was added along with TGF-β1 to LX2 cells, TGF-β1-induced expression of α-SMA and Snail was inhibited, but E-cadherin expression was elevated (Fig. 3G).

These results indicated that CHIR-99021 induced depolarization of HSCs via suppression of the EMT in a phenotypic-based 2D assay system using LX2 cells as well as in 3D MCHSs consisting of HSCs and HCC cells.

### CHIR-99021 suppresses DNMT3B expression in HSCs but not in HCC cells

Interestingly, other GSK3β inhibitors, except for CHIR-99021 and CHIR-98014, in the compound library could not induce an increase in MCHS size (Fig. 2D). Therefore, we examined the distinctive function of CHIR-99021, relative to other GSK3β inhibitors, in transactivating HSCs.

**Figure 4:**
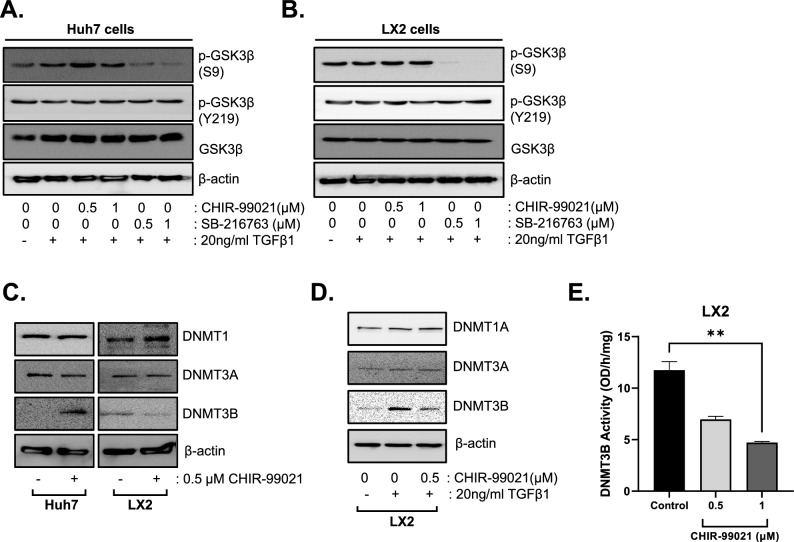
Suppression of DNMT3B in HSCs induced by CHIR-99021. (**A**,**B**) Huh7 (**A**) and LX2 (**B**) cells were treated with 20 ng/ml TGF-β1with or without CHIR-99021 and the GSK3 β inhibitor SB-216763. The expression levels of GSK3β and p-GSK3β (S9, Y219) were examined. C) Expression levels of DNMT3B induced by 0.5 µM CHIR-99021 in Huh7 and LX2 cells. (**D**) DNMT family proteins (DNMT1, DNMT3A, and DNMT3B) were examined in LX2 cells after treatment with 20 ng/ml TGF-β1 with or without 0.5 µM CHIR-99021. (**E**) DMNT3B activity was examined in LX2 cells after treatment with 0, 0.5 or 1uM of CHIR-99021. β-actin was used as a control.

GSK-3β phosphorylation at Ser9 by the PI3K/AKT signaling pathway leads to its inactivation, but GSK-3β phosphorylation at Tyr 216 induces activity. Unexpectedly, CHIR-99021 treatment of Huh7 (HCC cells) (Fig. 4A) and LX2 cells (HSCs) (Fig. 4B) did not alter phosphorylation of GSK-3β at Ser 9 or Tyr 216, unlike the effect of SB-216763. These results indicate that control of GSK-3β activation is not a major factor in depolarization of HSCs by CHIR-99021 treatment.

Next, we focused on the effect of CHIR-99021 on epigenetic regulatory gene expression in HSCs because inhibition of DNMTs prevents fibrogenic activation of HSCs. Among DNMTs, CHIR-99021 treatment inhibited DNMT3B expression in LX2 cells but not in Huh7 cells (Fig. 4C). Additionally, CHIR-99021 treatment downregulated DNMT3B protein expression in TGFβ-induced activated LX2 cells (Fig. 4D). Also, CHIR-99021 reduced the DNMT3B activity in LX2 cells (Fig. 4E).

### Suppression of DNMT3B effectively reverses HSC activation

**Figure 5:**
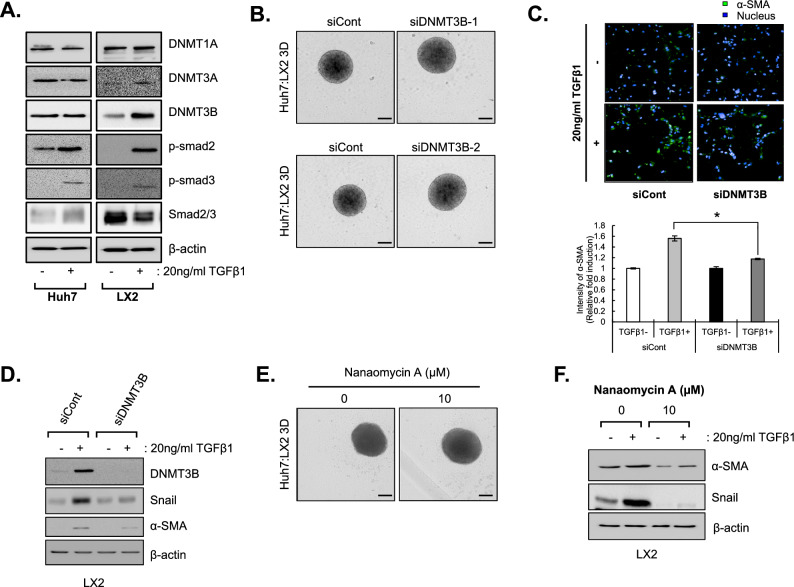
Reprogramming of HSCs by DNMT3B suppression. (**A**) Expression levels of DNMT family and Smad2/3-related proteins in Huh7 (left) and LX2 cells (right) were examined after treatment with 20 ng/ml TGF-β1. (**B**) Size and morphology of MCHSs composed of Huh7 and DNMT3B-deficient LX2 cells (in a 7:3 ratio) for 3 days. Two different siRNA used (upper: purchased from Dharmacon, lower: purchased from Bioneer). (**C**) DNMT3B-deficient LX2 cells were treated with or without 20 ng/ml TGF-β1 for 48 h. The morphology and α-SMA intensity were examined and calculated using an Operetta HTS system and Harmony software. (**D**) EMT-related proteins were examined in DNMT3B-deficient LX2 cells with or without 20 ng/ml TGF-β1 treatment. (**E**) Size and morphology of MCHSs composed of Huh7 and LX2 (in a 7:3 ratio) treated with 10 uM nanaomycin A for 3 days. (**F**) EMT-related proteins were examined in nanaomycin A treated LX2 cells with or without 20 ng/ml TGF- β1 treatment. β-actin was used as a control.

TGFβ treatment not only induces phosphorylation of p-Smad2/3 but also increases DNMT3B expression in HSCs, but not in hepatocytes (Fig. 5A).

We next explored the shape of MCHSs with Huh7 and DNMT3B-deficient LX2 cells via transfection of siDNMT3B into LX2 cells to investigate the effect of DNMT3B inhibition on MCHS compactness. Although MCHSs with Huh7 and normal LX2 cells exhibited enhanced spheroid compactness, MCHSs with Huh7 and DNMT3B-deficient LX2 cells showed loss of compactness (Fig. 5B).

To elucidate the cellular role of DNMT3B in the activation of HSCs during the development of liver fibrosis, we observed the morphological changes in HSCs induced by inhibition of DNMT3B with siDNMT3B after TGF-β1 treatment. Control LX2 cells had a polygonal shape with low α-SMA intensity. TGF-β1 treatment transformed the cells into a spindle shape and increased their α-SMA intensity, whereas siDNMT3B treatment reduced TGF-β1-induced protrusions and α-SMA intensity (Fig. 5C). When increases DNMT3B expression by TGFβ treatment was attenuated through transfection of siDNMT3B, TGF-β1-induced expression of α-SMA and Snail was inhibited in HSCs (Fig. 5D). Also, we treated MCHS with nanaomycin A, a specific DNMT3B inhibitor, to examine the same effect observed when siDNMT3B was used in MCHS. The results indicate a loss of compactness (Fig. 5E). Furthermore, upon confirming the activation of hepatic stellate cells by TGF- β1 by treating LX2 with nanaomycin, neither the related markers α-SMA nor snail were induced by TGF-β1 (Fig. 5F).

### CHIR-99021 markedly improves the efficacy of chemotherapeutic drugs against HCC

Our previous study showed that crosstalk between HCC and HSCs promoted sorafenib resistance in HCC cells. Here, we investigated whether modification of spheroid compactness, which was induced by depolarizing HSCs by pretreatment with CHIR-99021, could overcome resistance to anti-cancer therapy in MCHSs consisting of HSCs and HCC cells.

**Figure 6:**
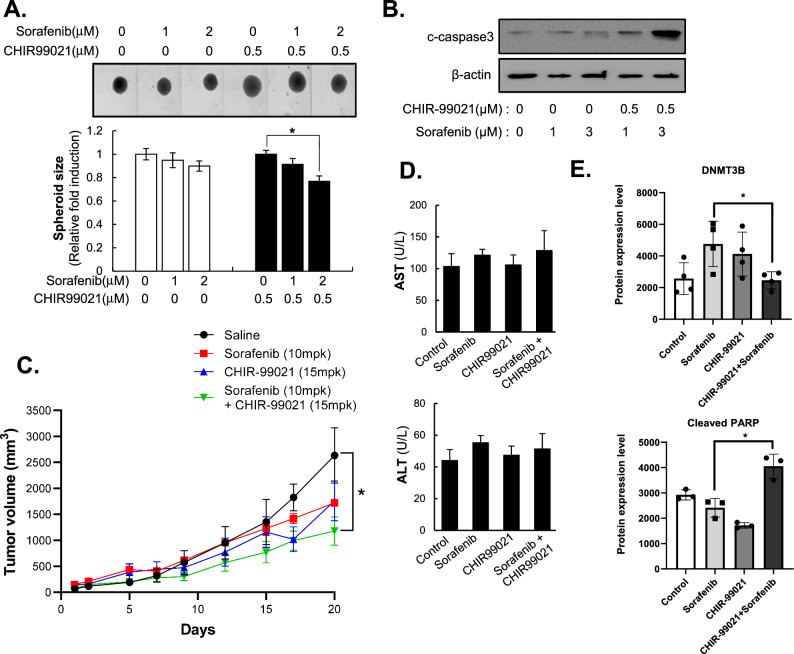
The efficacy of chemotherapeutic drugs against HCC was increased by CHIR-99021. (**A**) MCHS size was examined after treatment with 1 or 2 µM sorafenib with or without 0.5 µM CHIR-99021 for 2 days. Images (upper) were obtained using an Operetta HCS system, and MCHS sizes were calculated using Harmony software. (**B**) Cleaved-caspase 3, an apoptosis marker, was examined in MCHSs after treatment with 1 or 3 µM sorafenib with or without 0.5 µM CHIR-99021. (**C**) Tumor volume was measured for 3 weeks (1 week before and 2 weeks after drug administration) in the 10 mpk sorafenib, 15 mpk CHIR-99021, and 10 mpk sorafenib plus 15 mpk CHIR-99021 groups. (**D**) Liver toxicity was determined by examining mouse blood (AST; upper, ALT; lower) after completing the in vivo experiment. (**E**) Expression levels of DNMT3B and Cleaved PARP, an apoptosis marker, were calculated in xenografted tumor tissue. β-actin was used as a control.

An analysis of spheroid size showed that combination treatment with CHIR-99021 and sorafenib sufficiently induced a dose-dependent decrease in spheroid size in MCHSs composed of HSCs and HCC cells (Fig. 6A). Expression of an apoptosis-related marker, Cleaved-caspase 3, was measured following treatment with sorafenib, with or without CHIR-99021, in MCHSs composed of HSCs and HCC cells. Combination treatment with CHIR-99021 and sorafenib markedly increased Cleaved-caspase 3 in MCHSs composed of HSCs and HCC cells (Fig. 6B).

To determine whether CHIR-99021 could enhance the efficacy of anti-cancer therapies in an in vivo system, we generated a mouse xenograft model by injecting Huh7 cells into BALB/c-nu mice. Administration of 15 mpk CHIR-99021 produced only a subtle reduction of tumor growth, and 10 mpk sorafenib alone also induced tumor regression. In contrast, treatment with 10 mpk sorafenib plus 15 mpk CHIR-99021 significantly inhibited tumor volume compared with that in mice treated with sorafenib alone, without loss of body weight (Fig. 6C, Supplementary Fig. S2). No significant differences were observed in the aspartate aminotransferase (AST) and alanine aminotransferase (ALT) levels among CHIR-99021-treated mice, sorafenib-treated mice, and saline-treated mice (Fig. 6D). Western blot analysis of tumor tissues showed that treatment with sorafenib plus CHIR-99021 not only sufficiently inhibited DNMT3B expression but also increased Cleaved-PARP expression relative to that observed with sorafenib treatment alone in vivo (Fig. 6E). These results indicate that CHIR-99021 could sensitize cells to increase the efficacy of sorafenib treatment.

## Discussion

Many factors, such as hepatitis B, hepatitis C, alcohol use, and nonalcoholic liver disease, cause repeated and chronic inflammation of the liver^28^. Excess inflammation not only evokes necrosis and genetic alterations in hepatocytes but also leads to fibrosis and cirrhosis, which trigger microenvironmental alterations and may further contribute to the development of HCC^29,30^. Therefore, HCC is accompanied by inflammation, fibrosis, and cirrhosis. Currently, only two drug classes, including five angiogenesis inhibitors and two immune checkpoint inhibitors, are used to treat HCC and have FDA approval. Nevertheless, these single medications cannot entirely overcome this complex and deadly disease^31,32^.

Regarding systemic therapies, recently, the use of immunotherapy combined with angiogenesis inhibitors has been introduced, and the response rate is more satisfactory than that of conventional treatment. Hence, scientists think they can probably do more to make it even better. In this context, we examined the use of drugs in combination and in sequence and determined the best drugs to use in combination for HCC therapy, which is an area that requires further research.

Most liver cancer patients have liver cirrhosis, which is a symptom of the hardening and contracting of liver tissue due to liver fibrosis. We are developing a liver fibrosis treatment to help restore hardened liver tissue to its normal state and maximize the absorption rate and therapeutic effects of anti-cancer drugs. To this end, we have focused on the development of treatments for liver cancer and liver fibrosis through the development of a unique 3D MCTS model for multiplexed screening to optimize early identification of drugs likely to be active in the diseased liver^11,12,26,27^. In particular, MCTSs consisting of HSCs and HCC cells were established and used for drug discovery to improve the therapeutic efficacy of conventional anti-cancer drugs, thereby inducing the reprogramming of activated HSCs in the tumor microenvironment.

Activated HSCs transform into myofibroblast-like cells to promote fibrosis in response to liver injury or chronic inflammation, leading to cirrhosis and HCC. Therefore, it is imperative to study the control mechanism of activated HSCs to develop new drugs for more effective treatment of HCC and to augment current therapies and increase their success^14,33,34^.

Surprisingly, CHIR-99021, a GSK-3β inhibitor, was selected as a HIT compound when screening for drugs that could reverse HSC activation in MCTS. GSK-3β has attracted attention as a promising new treatment for tissue fibrosis, and many studies have indicated that GSK3β inhibition contributes to HSC activation and liver fibrosis^35^. However, we showed that CHIR-99021 acts specifically on HSCs in the tumor environment and exerts anti-fibrotic effects by regulating DNMT3B activity rather than GSK3β activity. Recently, DNMTs have been recognized as regulators of fibrogenesis because they can reprogram the HSC transcriptome^36,37^. For example, 5-AzadC, a DNMT inhibitor, can suppress HSC transdifferentiation, providing further evidence of the importance of DNA methylation in fibrosis^38^. However, the effect of DNMT3B on HSC activation during fibrogenesis has rarely been studied.

In terms of its anti-fibrotic effects, CHIR-99021 could effectively control HSC activation, and inhibition of DNMT3B by CHIR-99021 increased sensitivity to sorafenib in MCTSs in vitro as well as in MCTS xenograft mice. These results suggest a therapeutic potential for CHIR-99021 as part of a combination therapy. Additionally, future development of DNMT3B inhibition as a therapeutic strategy for HCC will require further study into the selection of patients based on molecular vulnerabilities.

### Supplementary Information

Supplementary Figures.

## Data Availability

The datasets used and/or analysed during the current study available from the corresponding author on reasonable request.
