# Supplementary material for: Inhibition of DNMT3B expression in activated hepatic stellate cells overcomes chemoresistance in the tumor microenvironment of hepatocellular carcinoma
Source: Sci Rep. 2024 Jan 2;14:115. doi: 10.1038/s41598-023-50680-6 (PMC10761987; doi:10.1038/s41598-023-50680-6)

**Supplementary Figure**


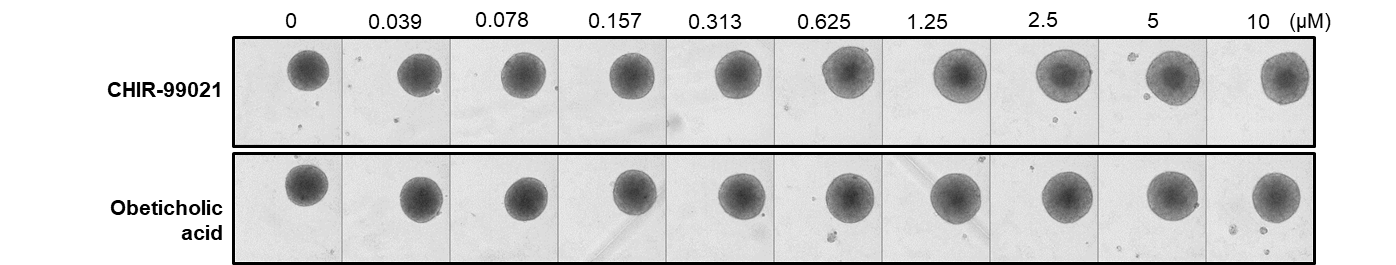


**Supplementary Figure 1. The effects of CHIR-99021 and Obeticholic acid in MCHS.**

**Supplementary Figure 2. The body weight of mouse treated with saline, Sorafenib, CHIR-99021, and Sorafenib and CHIR-99021 combination for 20days.**


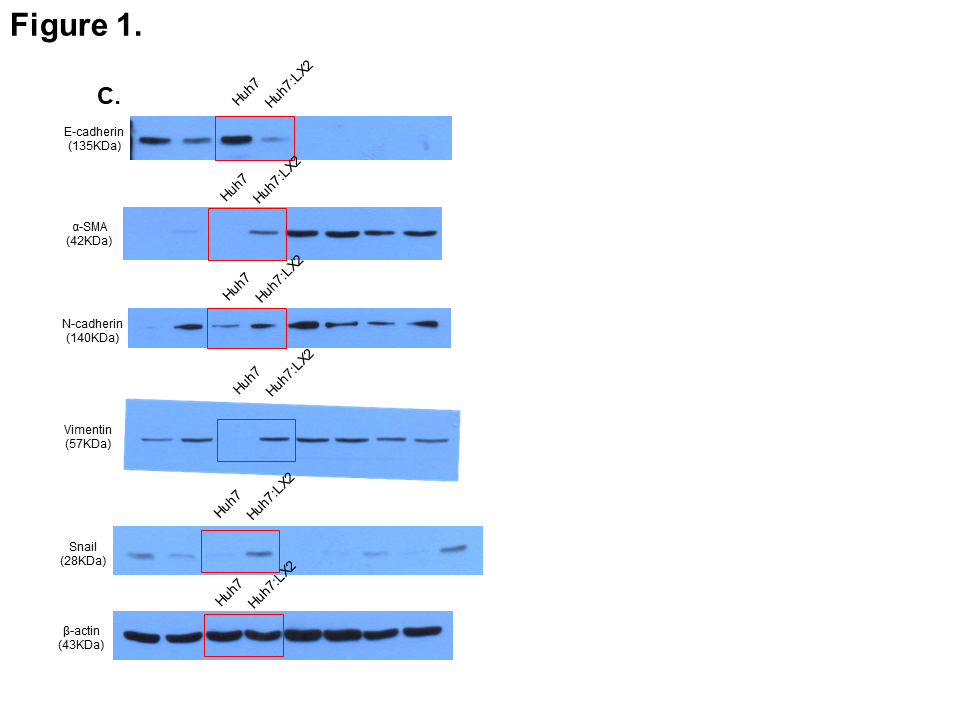

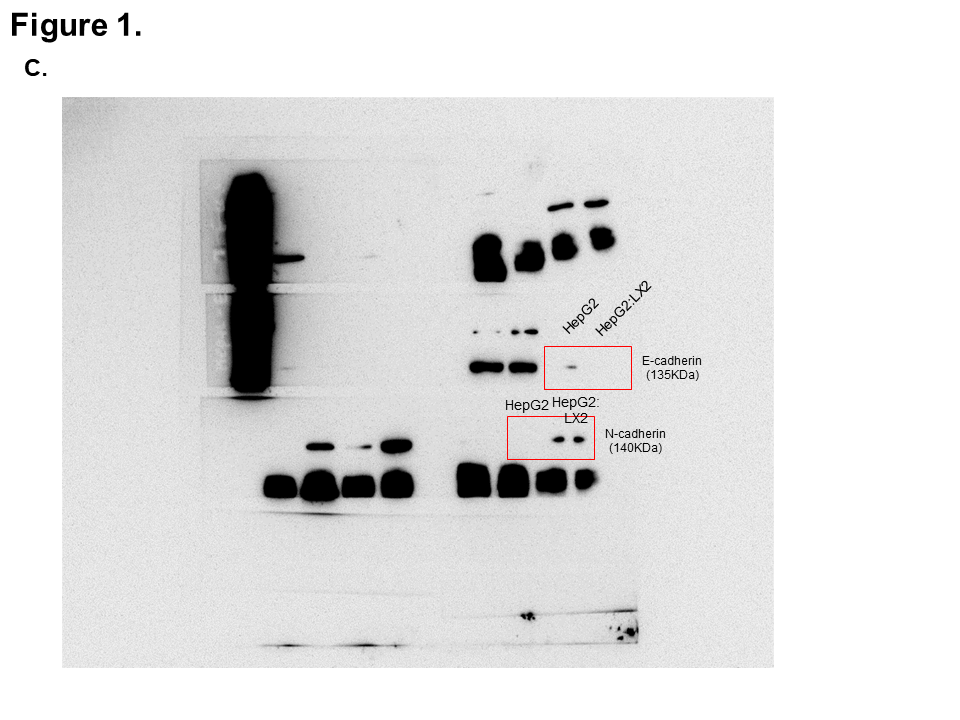

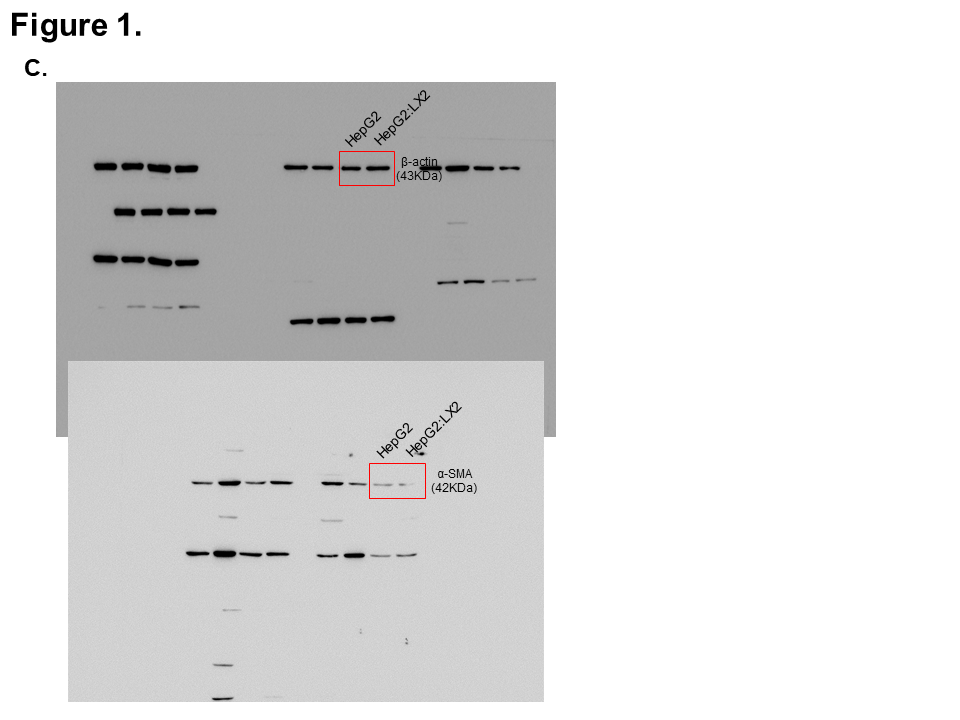

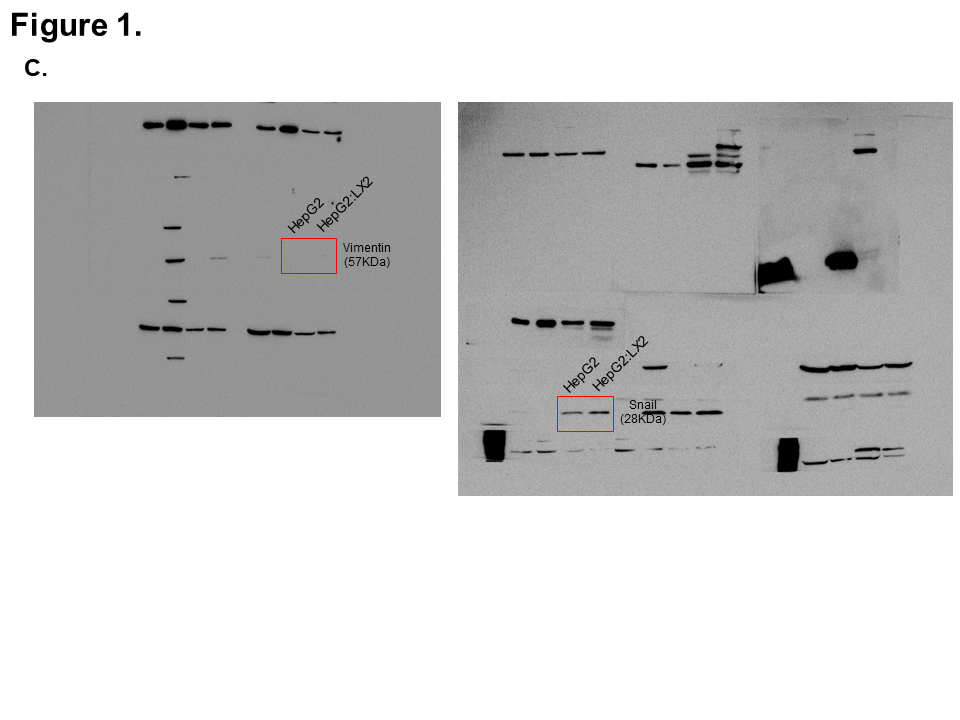

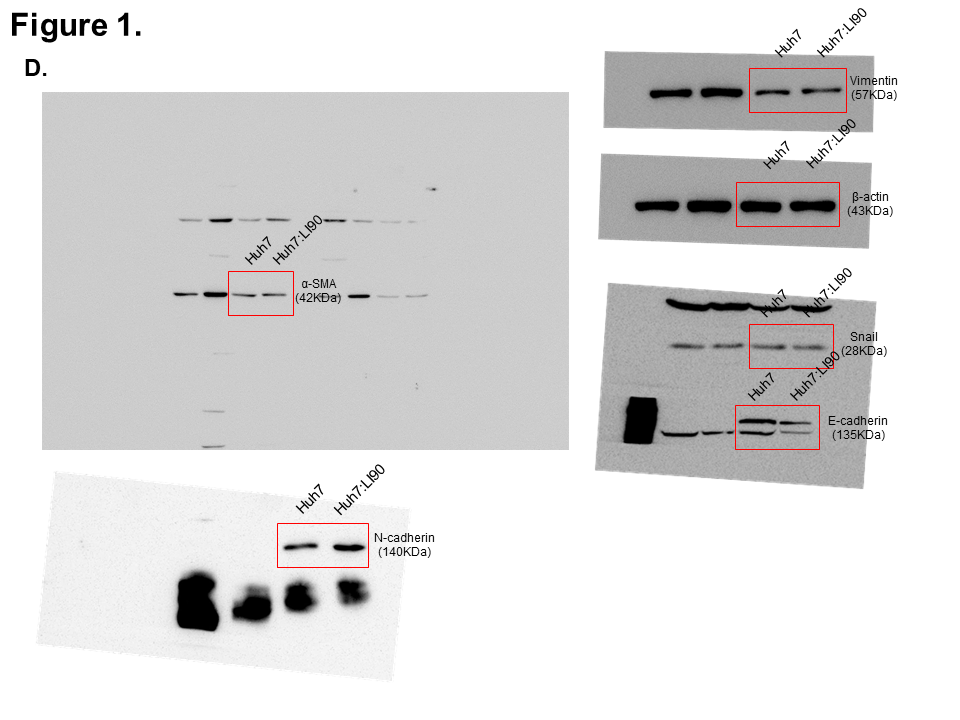

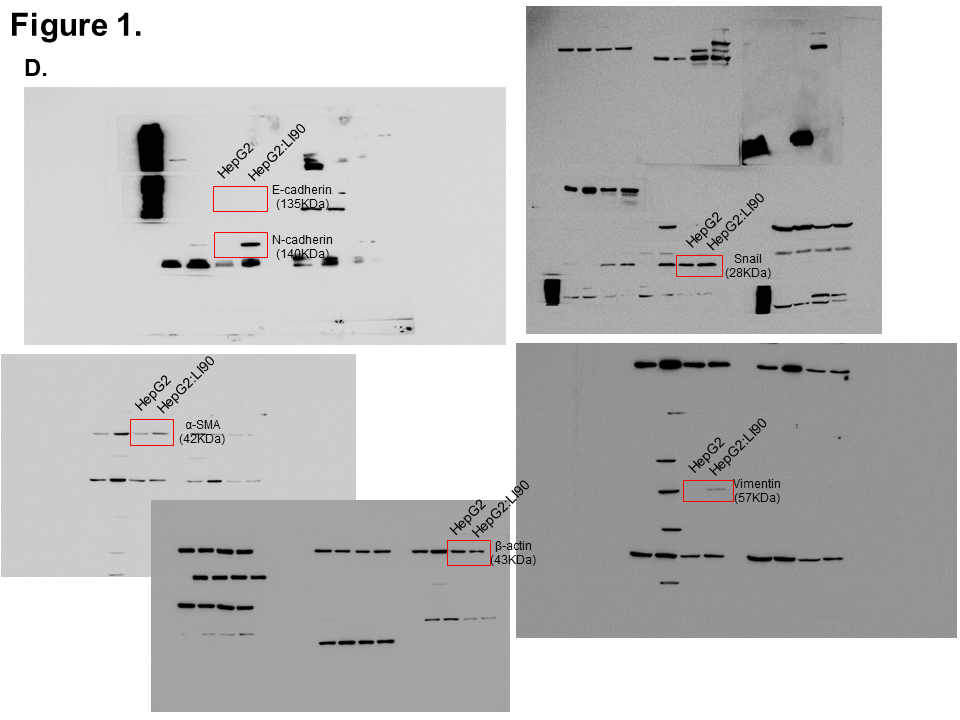

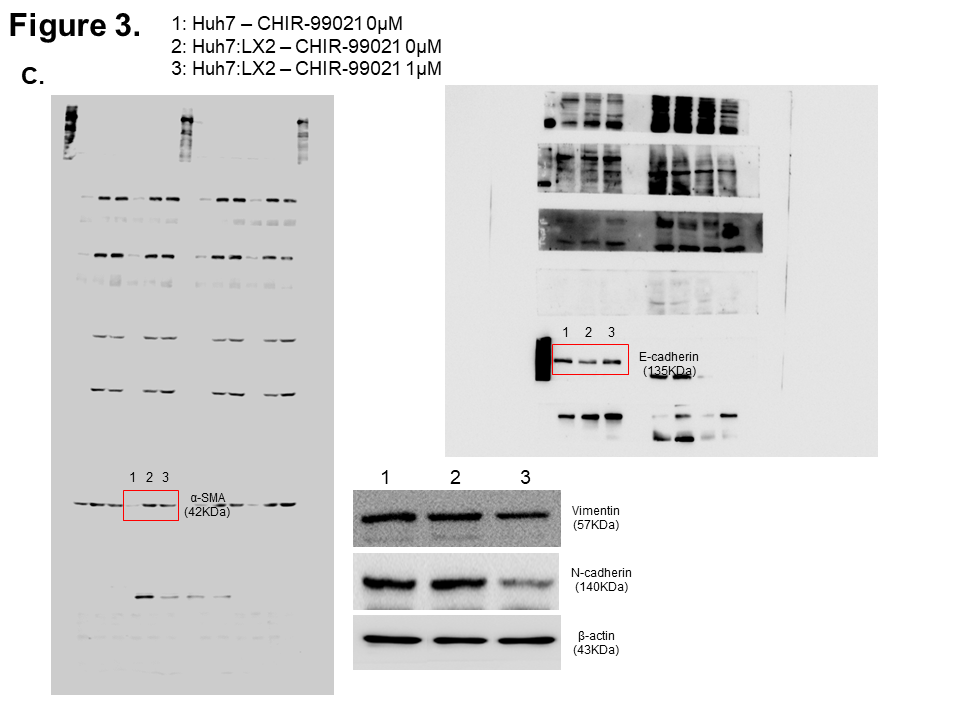

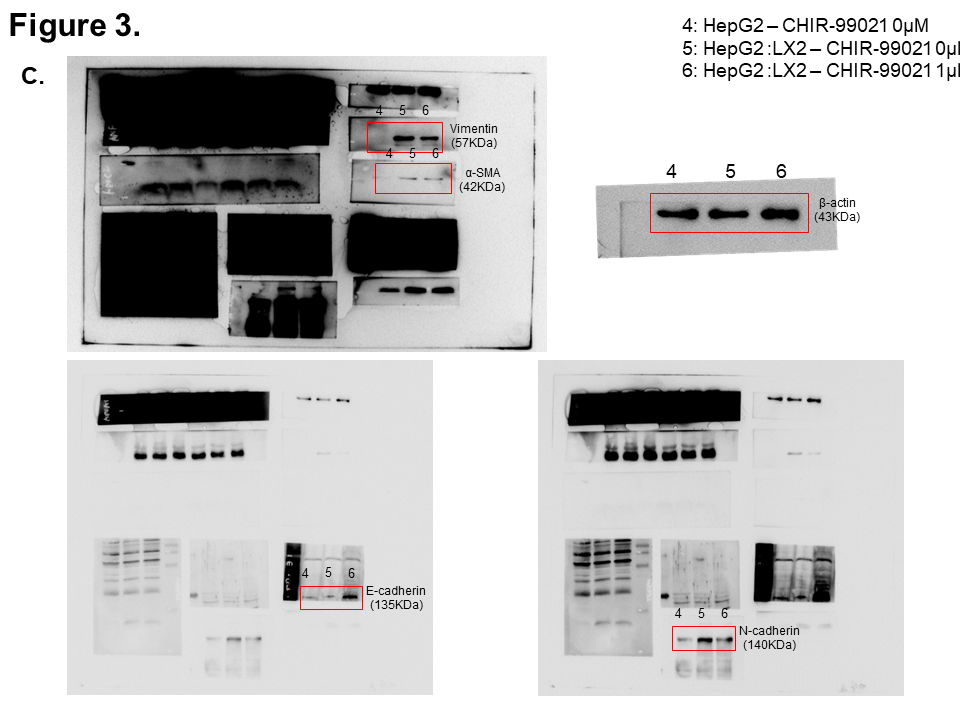

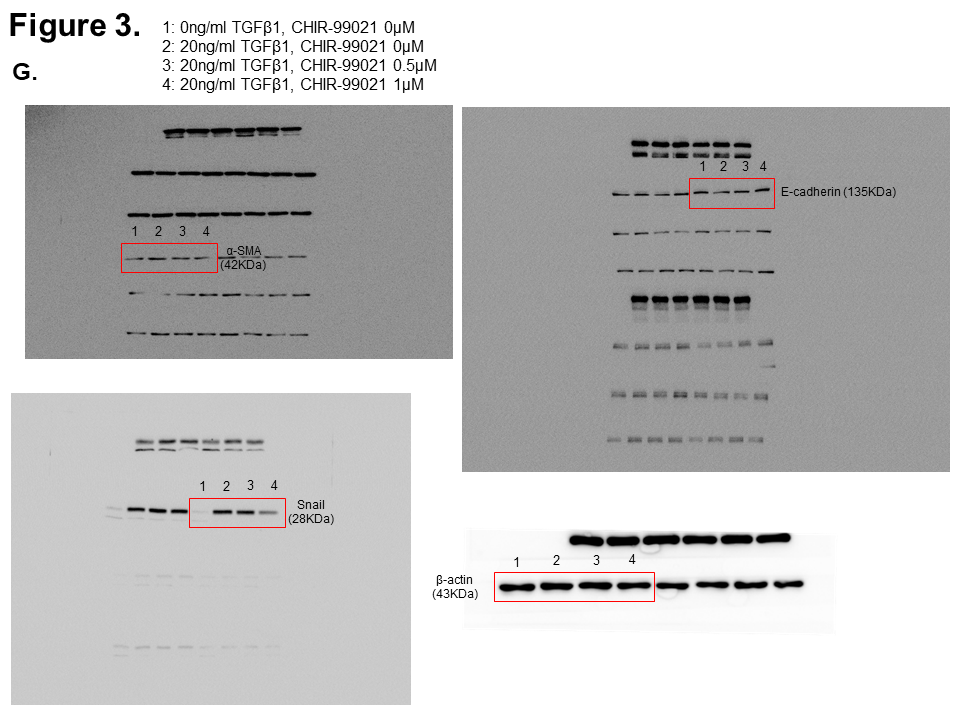

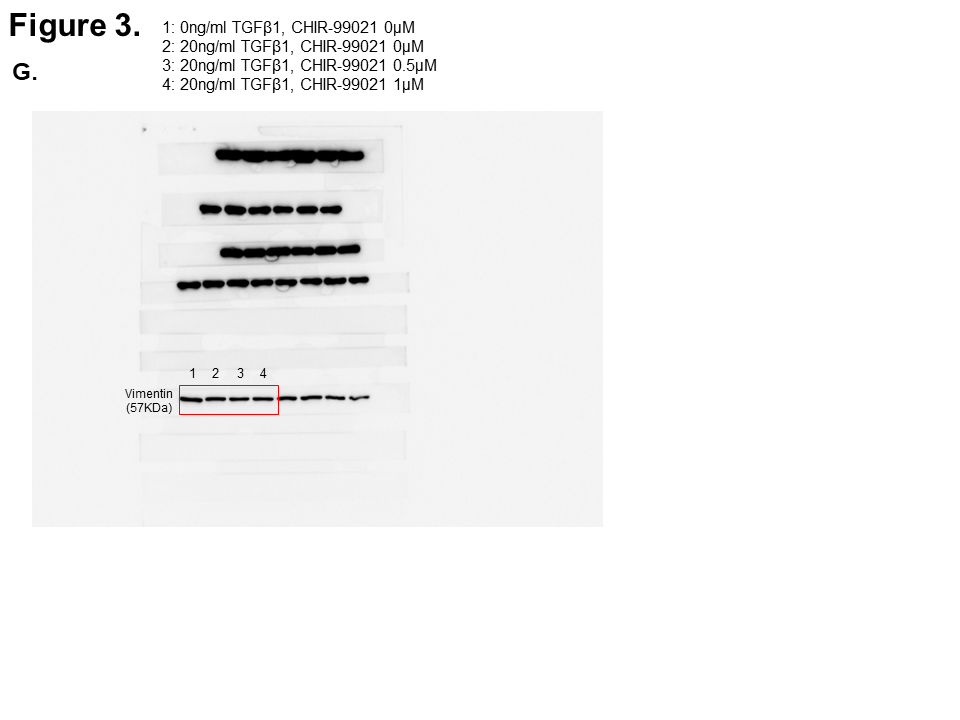

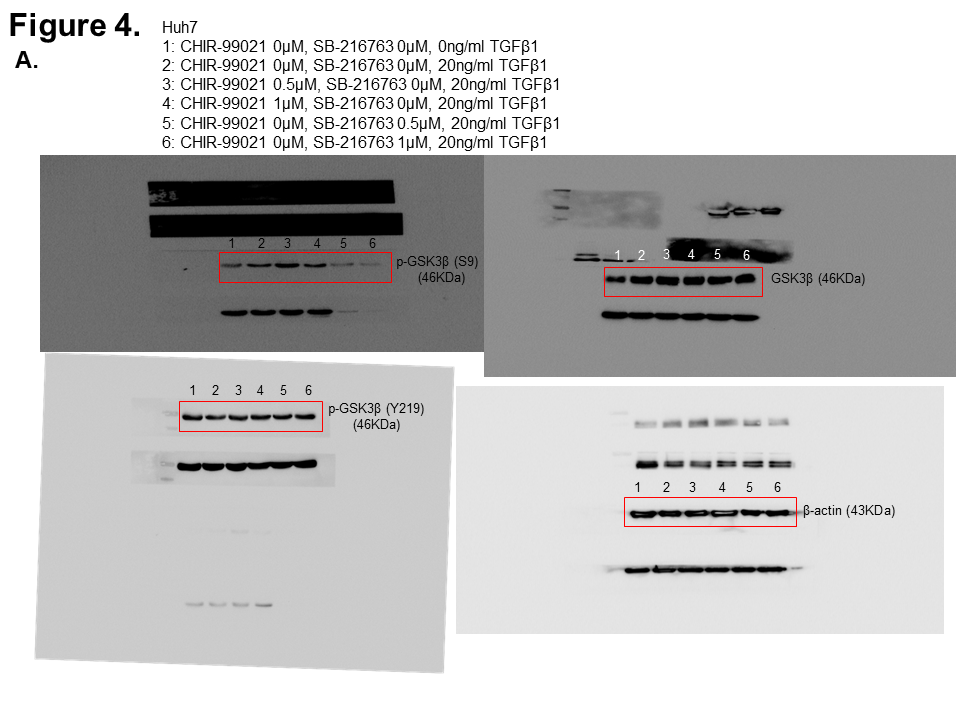

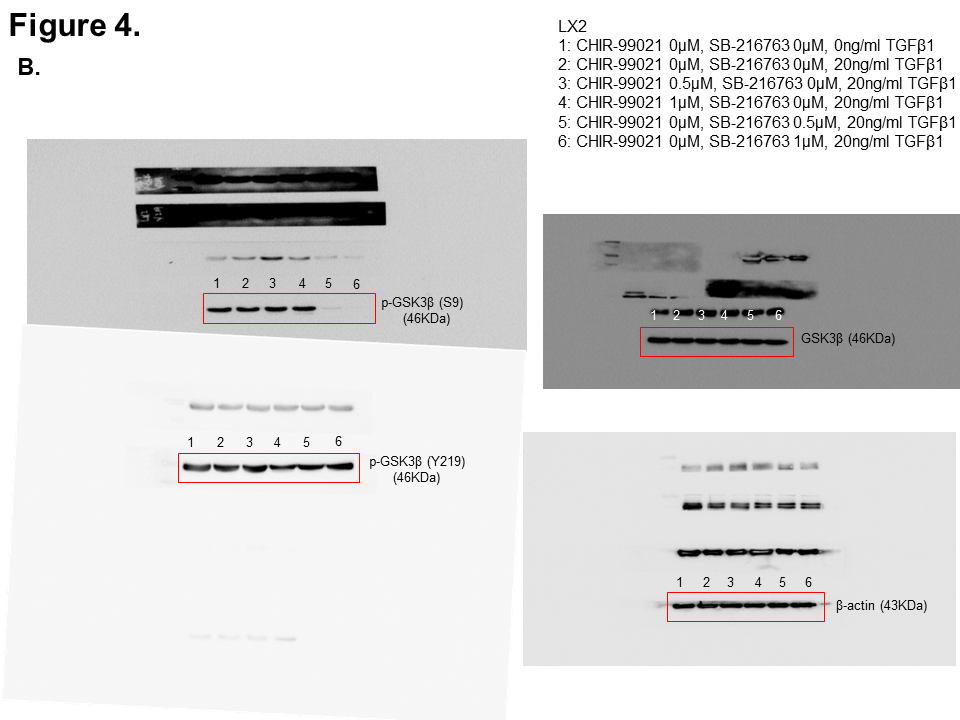

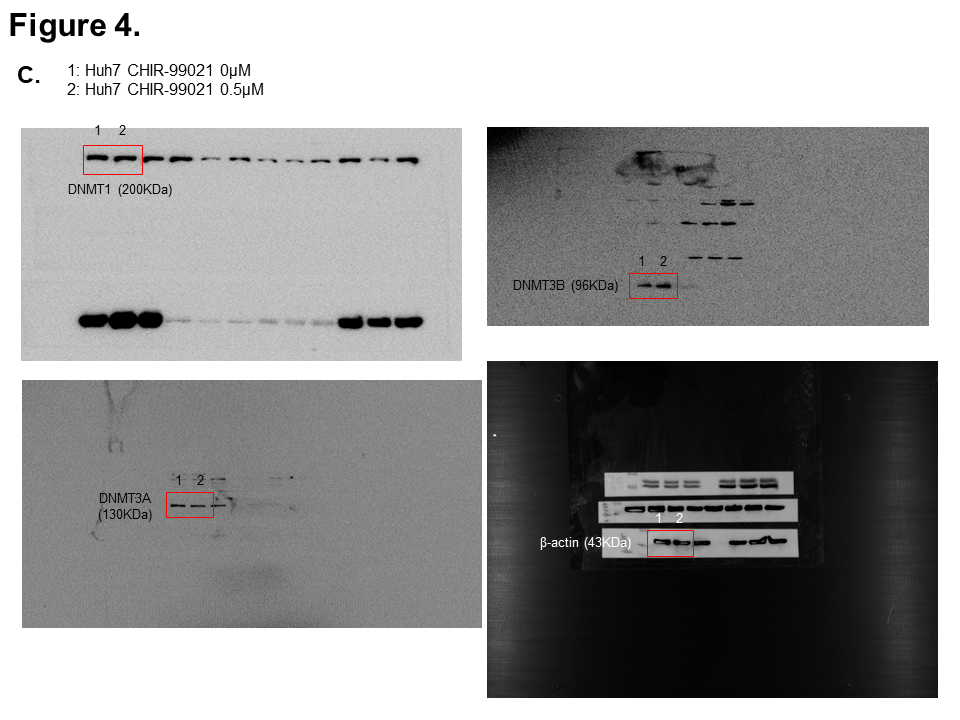

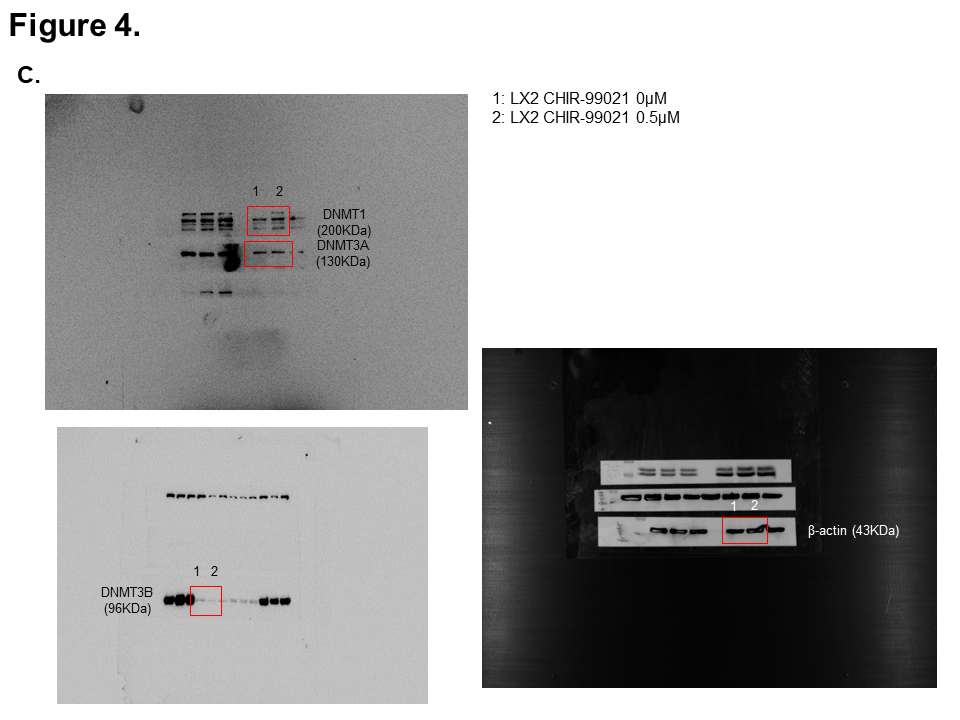

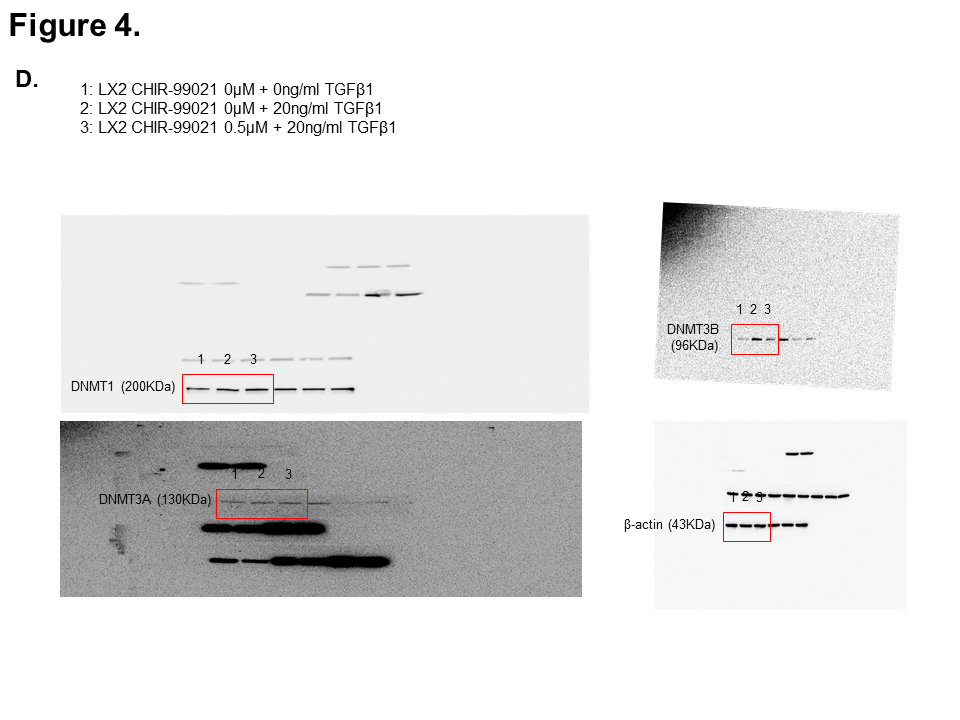

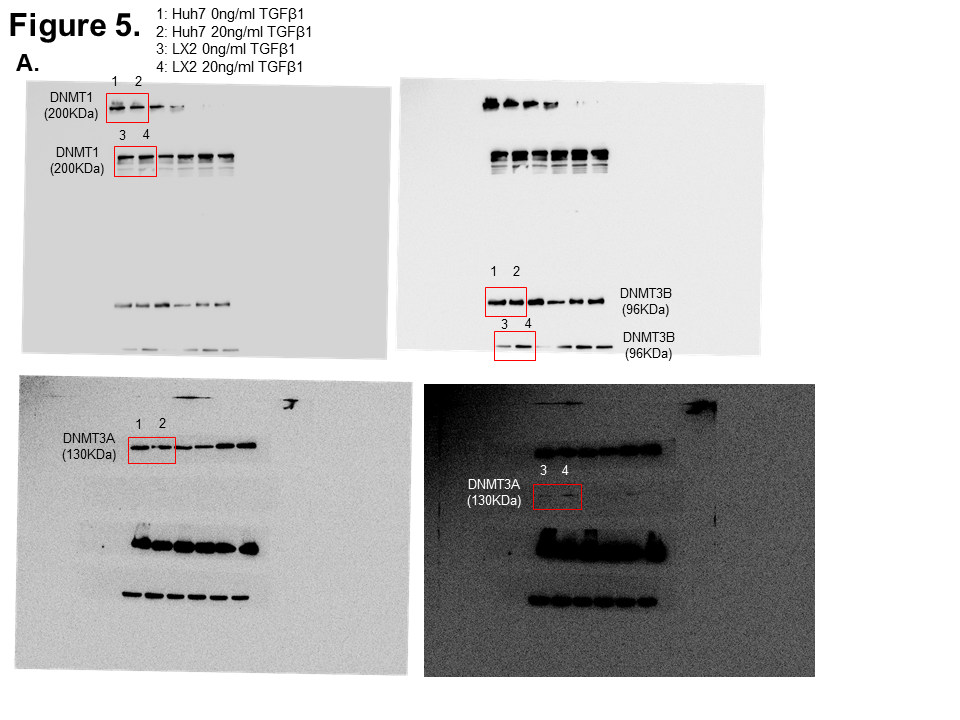

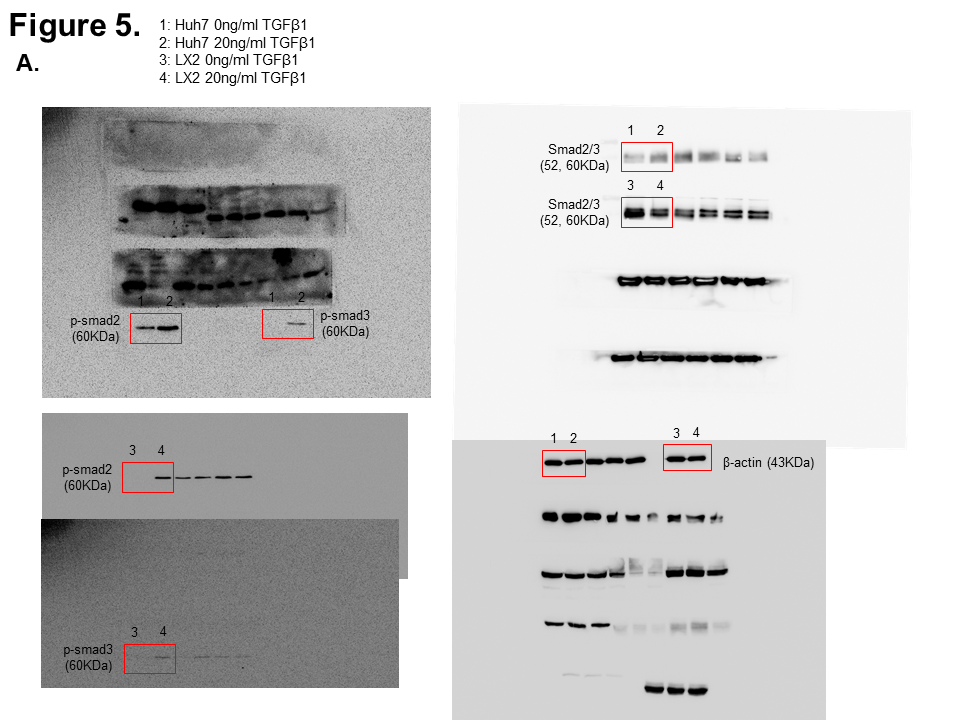

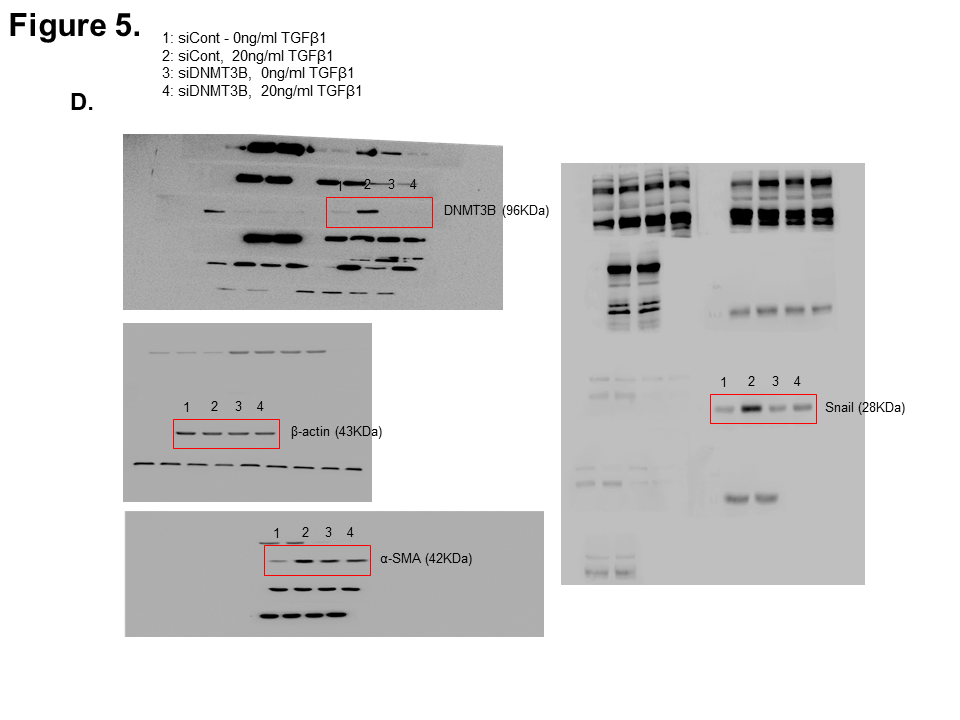

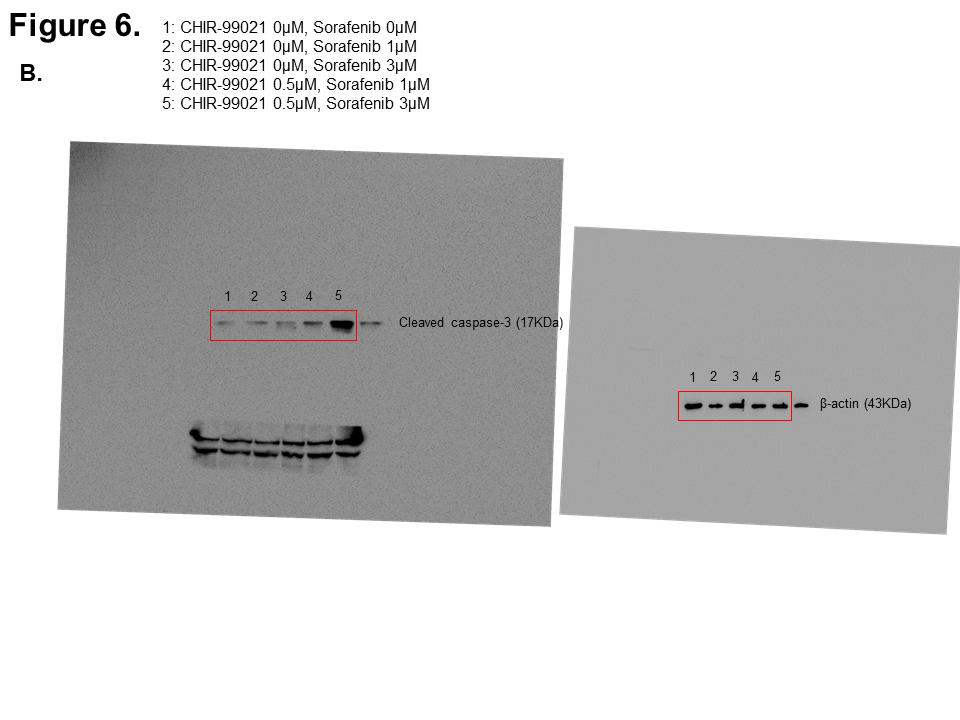

Supplement: Supplementary file 1 — Supplementary Figures. [file 41598_2023_50680_MOESM1_ESM.docx]
